# Taurolithocholic acid alleviates hepatic glycolipid dysregulation in low-birth-weight piglets by activating HNF4α and FXR

**DOI:** 10.1186/s40104-026-01474-z

**Published:** 2026-08-05

**Authors:** Tianle Gao, Hairong Liang, Meizheng Li, Rui Zhou, Li Wang, Ran Li, Lingjie Huang, Yong Zhuo, Shengyu Xu, Yan Lin, Bin Feng, Jian Li, Hua Li, Lun Hua, Lianqiang Che, De Wu, Liang Hu, Zhengfeng Fang

**Affiliations:** 1 Key Laboratory for Animal Disease-Resistance Nutrition of China Ministry of Education, Animal Nutrition Institute, Sichuan Agricultural University, 211 Huimin Road, Wenjiang District, Chengdu, 611130 China; 2 Dekon Food and Agriculture Group, Chengdu, China; 3 Key Laboratory of Agricultural Product Processing and Nutrition Health (Co-Construction By Ministry and Province), Ministry of Agriculture and Rural Affairs, College of Food Science, Sichuan Agricultural University, Ya’an, 625014 China

**Keywords:** Bile acid, FXR, HNF4α, Low birth weight, Piglet, Taurolithocholic acid

## Abstract

**Background:**

Low-birth-weight (LBW) piglets often exhibit glycolipid metabolic disorders at birth, which severely impair their postnatal growth and survival. Bile acids (BAs) act as signaling molecules that participate in the regulation of glycolipid metabolism. However, whether the hepatic metabolic abnormalities observed in LBW piglets are associated with altered BA metabolism remains largely unclear. Thus, using naturally occurring LBW fetal pigs, neonatal piglets, and hepatic cell lines as models, the present study aimed to elucidate the association between BA homeostasis and hepatic glycolipid metabolism and to further reveal the underlying molecular mechanisms through integrated analyses of BA-targeted metabolomics, 16S rRNA gene sequencing, and molecular docking.

**Results:**

Compared with normal-birth-weight (NBW) fetal pigs, LBW fetal pigs exhibited a marked reduction in hepatic glycogen storage accompanied by excessive lipid accumulation. As key nuclear receptors governing glycolipid metabolism, farnesoid X receptor (FXR) and hepatocyte nuclear factor 4α (HNF4α) were significantly down-regulated in the liver of LBW fetal pigs at both the transcriptional and protein levels, which was coupled with impaired glycogen synthetic capacity and lipolytic capacity in these fetal pigs. Targeted BA metabolomic analysis revealed a profound alteration in the hepatic BA profile of LBW fetal pigs, characterized by an increased proportion of secondary BAs. Notably, the hepatic level of taurolithocholic acid (TLCA) was markedly decreased in LBW fetal pigs. Further analyses demonstrated that critical processes of BA metabolism, including synthesis, transport, detoxification and conjugation, were impaired in LBW fetal pigs, along with disrupted endogenous TLCA biosynthesis. Mechanistically, molecular docking results suggested that TLCA might act as a potential agonist of FXR and HNF4α. In vitro assays confirmed that TLCA modulated hepatic glycolipid metabolism by activating FXR and HNF4α. More importantly, in vivo studies indicated that exogenous supplementation of TLCA significantly ameliorated hepatic glycolipid metabolism and improved overall liver function in LBW neonatal piglets.

**Conclusions:**

These findings reveal crosstalk between hepatic glucose-lipid and BA metabolism via HNF4α and FXR, providing potential nutritional strategies to improve liver health in LBW piglets and a theoretical basis for using BAs as feed additives in pig production.

**Supplementary Information:**

The online version contains supplementary material available at 10.1186/s40104-026-01474-z.

## Background

In recent years, bile acids (BAs) have emerged as key biomolecules in regulating glucose and lipid metabolism, with their homeostasis influenced by genetic factors, dietary composition, and lifestyle [1–3]. Our previous study in a weaned piglet model demonstrated that insufficient nutrient intake perturbs BA metabolism through alterations in gut microbial composition, thereby disrupting the gut–liver farnesoid X receptor (FXR) signaling axis [4]. By contrast, low-birth-weight (LBW) piglets, which frequently result from intrauterine growth restriction (IUGR), are widely regarded as a model of “fetal undernutrition” primarily arising from impaired maternal–placental–fetal nutrient transfer [5, 6]. LBW is not only associated with increased perinatal morbidity and mortality but also linked to a heightened risk of metabolic syndrome in adulthood, including obesity, type 2 diabetes, cardiovascular diseases, and non-alcoholic fatty liver disease (NAFLD) [7]. The liver is the central organ for glucose and lipid metabolism, and LBW neonatal piglets are born with hepatic dysfunction, characterized by depleted glycogen stores and lipid accumulation, which severely restricts growth and development [8, 9]. A recent study demonstrated that tauroursodeoxycholic acid (TUDCA) can ameliorate high-fat diet-induced NAFLD by regulating gut microbial composition and BA metabolism [10]. However, it remains unclear whether BA metabolism is involved in regulating LBW-associated hepatic glucose and lipid metabolic disorders.

Hepatocyte nuclear factor 4α (HNF4α), a hepatic nuclear receptor, maintains liver homeostasis by regulating hepatocyte proliferation, differentiation, and the expression of key genes involved in glucose and lipid metabolism [11–13]. Overexpression of hepatic HNF4α promotes lipolysis by enhancing fatty acid oxidation and very-low-density lipoprotein (VLDL) secretion, preventing excessive hepatic lipid accumulation [12]. Mice with hepatic HNF4α knockout show reduced fasting blood glucose and marked alterations in the expression of hepatic genes involved in glycolysis, gluconeogenesis, and glycogen metabolism [14]. As a major BA receptor in the liver, FXR regulates BA synthesis and transport and contributes to lipid and glucose homeostasis through crosstalk with factors such as HNF4α [1, 15]. Notably, studies in fetal mouse livers have shown that liver-specific HNF4α knockout significantly reduces FXR expression [16], suggesting a regulatory relationship between these nuclear receptors during hepatic development. Activation of FXR promotes fatty acid β-oxidation by upregulating hepatic PPARα [17]. FXR activation also reduces plasma triglyceride levels by inhibiting the hepatic transcription factor SREBP-1c and its lipogenic target genes [18]. FXR knockout mice present with elevated serum free fatty acids, triglycerides, and high-density lipoprotein cholesterol, together with hyperglycemia, impaired glucose tolerance, and markedly reduced insulin signaling in the liver and muscle [19, 20]. However, despite the established roles of HNF4α and FXR in hepatic metabolism and their confirmed crosstalk, it remains unclear whether alterations in BA composition and metabolism in LBW fetal livers contribute to glucose and lipid dyshomeostasis by regulating the activity or expression of FXR and HNF4α.

Given that LBW fetal pigs have been confirmed to exhibit hepatic glucose and lipid metabolic disorders, and the activity of FXR and HNF4α is dependent on activation by endogenous BA ligands [21, 22], we hypothesize that abnormalities in BA composition or metabolism in the livers of LBW fetal pigs may affect the functionality of FXR and HNF4α, thereby exacerbating hepatic glucose and lipid homeostasis imbalance. To test this hypothesis, we used naturally occurring LBW fetal pigs, neonatal piglets, and the Hepa 1-6 cell line as in vivo and in vitro models to investigate whether BA can ameliorate hepatic glucose and lipid metabolic disorders by activating FXR and HNF4α. This study aims to provide a theoretical basis and intervention strategies for targeting BA metabolism to regulate liver function and correct hepatic glucose and lipid metabolic abnormalities in LBW neonates.

## Materials and methods

### Experimental design and animal feeding management

The study protocol was approved by the Animal Care and Use Committee of the Animal Nutrition Institute (2023082114010) at Sichuan Agricultural University, and it adhered to the guidelines outlined in the National Research Council's Guide for the Care and Use of Laboratory Animals. Pregnant sows of the Landrace and Yorkshire breeds were chosen from the swine farm of the Animal Nutrition Institute at Sichuan Agricultural University, located in Ya'an, China. Sows were housed in individual pregnancy crates (2.0 m × 0.6 m) from d 0 of gestation. On d 107, sows were transferred to farrowing cages (2.0 m × 2.5 m). The temperature and humidity of the gestation house were controlled at 16–25 °C and 50%–70%, respectively. Gestating and lactating sows’ basal diet (Additional file 1: Table S1) was formulated to meet or exceed the nutrient requirements for both gestating and lactating animals (NRC, 2012) [23]. The feeding program for sows during gestation and lactation was consistent with our previous description [24]. All sows were immunized according to the standards of farm breeding procedures.

#### Animal Exp. 1: LBW induces hepatic glucose-lipid metabolic dysregulation in fetal pigs

LBW (0.84–1.02 kg, *n* = 6) and NBW (1.72–2.12 kg, *n* = 6) fetal pigs were harvested via cesarean section from four third-parity sows on gestational d 112. The average litter size of sows was 13.25 ± 2.98. To minimize litter effects, each sow contributed ≥ 1 qualified fetal pig to both groups. LBW fetal pigs (< 1.1 kg) were classified as previously described [25]. Cesarean sections followed established protocols [26]. All sows were euthanized immediately after the procedure.

#### Animal Exp. 2: TLCA ameliorates hepatic glucose-lipid homeostasis in LBW neonatal pigs via HNF4α and FXR

Thirty-two neonatal pigs were derived from 8 fourth-parity sows (average litter size was 12.40 ± 2.08) of the same genetic background, with each sow contributing 1 NBW pig and 3 LBW pigs. The 8 NBW pigs (1.34–1.73 kg) served as positive controls. The remaining 24 LBW (0.69–1.03 kg) pigs were randomized into three groups using weight-stratified randomization (*n* = 8 per group): LBW control, LBW + taurolithocholic acid (TLCA, 2 mg/kg BW), and LBW + chenodeoxycholic acid (CDCA, 30 mg/kg BW). From postnatal d 0, all neonatal pigs were evenly fostered to 4 healthy, fourth-parity lactating sows at the same lactation stage, with each sow receiving 2 pigs from each of the four groups (2 NBW, 2 LBW, 2 LBW + TLCA, 2 LBW + CDCA). On postnatal d 28, 6 pigs with body weights close to the group mean were randomly selected from each group for euthanasia and sampling. TLCA dosage was based on a prior report [27], while that of CDCA followed our preliminary study in newborn pigs [28]. Prior to administration, the two BAs were dissolved in a solvent composed of 10% (v/v) dimethyl sulfoxide (DMSO), 40% (v/v) polyethylene glycol (PEG), 5% (v/v) Tween-80, and 45% (v/v) normal saline. After dissolution, the solution was warmed to 37–40 °C and administered orally daily from postnatal d (PND) 1 to PND 27 at 14:00 using a syringe, at a volume of 5 mL per dose. Pigs in the NBW control group and LBW control group were both given an equal volume of the vehicle. During week 1 of the experiment, routine husbandry practices were implemented for the pigs, including iron supplementation and pseudorabies vaccination. Simultaneously, the housing temperature was maintained at 28–30 °C, and the humidity was kept at 60%.

### Sample collection

In Exp. 1, amniotic fluid (5 mL) was collected from each fetal pig using a sterile syringe, and fetal body weight was recorded. After identifying the selected fetal pigs, 2 mL of blood was obtained from the anterior vena cava. The fetal pigs were then euthanized, and liver tissues were harvested for subsequent biochemical and histological analysis. Colonic contents were collected in sterile 2-mL tubes and immediately stored at −80 °C until subsequent 16S rRNA gene sequencing analysis. In Exp. 2, on d 28 of the trial, six suckling pigs with body weights close to the group mean were randomly selected per group, and 5 mL of blood was collected from each piglet. Following blood collection, the piglets were euthanized, and liver tissues were harvested for biochemical and histological analysis. Amniotic fluid samples were immediately stored at −80 °C until analysis. Blood samples were kept at room temperature for 30 min after collection, followed by centrifugation at 3,500 × *g* for 15 min at 4 °C. The resulting serum was then stored at −80 °C for further analysis. Liver tissues for biochemical assays were flash-frozen in liquid nitrogen and stored at −80 °C, while those for histological analysis were fixed in 4% paraformaldehyde until further processing.

### Biochemical assays

Serum concentrations of alkaline phosphatase (ALP), γ-glutamyl transferase (GGT), non-esterified fatty acids (NEFA), triglycerides (TG), total cholesterol (TC), high-density lipoprotein cholesterol (HDL-C), low-density lipoprotein cholesterol (LDL-C), total bilirubin (TBIL), direct bilirubin (DBIL), aspartate transaminase (AST), alanine transaminase (ALT) and total BA were measured using an automatic biochemical analyzer (Hitachi 3100, Tokyo, Japan). The levels of sulfotransferase 2A1 (SULT2A1) in liver tissue and serum, interleukin-6 (IL-6), interleukin-1β (IL-1β), tumor necrosis factor-α (TNF-α) and solute carrier family 27 member 5 (SLC27A5) in liver tissue, as well as taurine and glycine in serum, liver, and amniotic fluid, were quantified using commercial ELISA kits (Jiangsu Enzyme-linked Immunosorbent Assay Biotechnology Co., Ltd., Jiangsu, China). All procedures were performed strictly in accordance with the manufacturer’s instructions.

### Histology staining and analysis

Liver tissue blocks (~0.3 cm^3^) were excised from the central region of the left hepatic lobe. Specimens were fixed in 4% (w/v) paraformaldehyde (PFA) and embedded in either paraffin or optimal cutting temperature (OCT) compound. Paraffin-embedded Sects. (4 μm thick) were stained with hematoxylin and eosin (H&E) or periodic acid-Schiff (PAS), while OCT-embedded samples were rapidly frozen, sectioned at 8 μm, and stained with Oil Red O (ORO; Leica Microsystems, Shanghai, China). Sections were randomly selected for examination. Brightfield microscopy (Nikon TS100, Tokyo, Japan) equipped with a charge-coupled device (CCD) camera and NIS-Elements F3.2 software (Nikon) was used to capture images. Each section was analyzed at 200 × magnification, with at least three randomly selected fields per section assessed. Lipid droplet content and hepatic glycogen levels were quantified using Image Pro Plus 6.0 software (Media Cybernetics, Rockville, MD, USA).

### Determination of fatty acid composition

Hepatic fatty acid profiles were determined with minor modifications to a previously described method [29]. Briefly, approximately 100 mg of freeze-dried liver tissue was homogenized in 3 mL chloroform-methanol-water (8:4:3, v/v/v) in glass tubes. After vortexing for 1 min and phase separation, the chloroform layer was collected and evaporated under a stream of nitrogen. The lipid residue was saponified with 2 mL of 0.5 mol/L methanolic KOH at 50 °C for 10 min. After cooling, the mixture was extracted with 1 mL n-hexane containing 0.05 g/L BHT and 2 mL saturated NaCl, followed by centrifugation at 1,000 × *g* for 5 min at 25 °C. At least 600 μL of the hexane supernatant was analyzed by gas chromatography (CP-3800, Varian, USA) equipped with an SP-2560 capillary column (100 m × 0.25 mm ID, 0.20 μm film thickness). Fatty acid composition is expressed as the relative percentage of total fatty acids.

### Analysis of BA profiles

Approximately 10 mg of liver tissue was homogenized in a 2-mL centrifuge tube containing an extraction solvent. The homogenate underwent a series of centrifugation, freeze-drying, and reconstitution steps. The resulting supernatant was then analyzed using an ACQUITY ultraperformance liquid chromatography (UPLC) system (Waters, Milford, MA, USA) coupled with triple-quadrupole mass spectrometry (MS/MS) for BA quantification. The analytical method employed was adapted from previously established protocols [30].

### 16S rRNA gene sequencing

Genomic DNA of the microbial community was extracted from colonic content using QIAamp DNA Stool Mini kits (Qiagen Inc., Hilden, Germany) in accordance with the manufacturer’s standard operating procedures. After extraction, DNA integrity was confirmed by 1% agarose gel electrophoresis, and both its concentration and purity were determined with a NanoDrop 2000 spectrophotometer (Thermo Scientific, Waltham, MA, USA). According to the aforementioned quantification data, all DNA samples were normalized to a concentration of 1 ng/μL with sterile deionized water and served as templates for polymerase chain reaction (PCR) amplification. Raw sequence data were processed utilizing Uparse software (Version 7.0.1001). The hypervariable V3–V4 regions of the 16S rRNA gene were PCR-amplified with the specific primer set 341 F (5′-CCTAYGGGRBGCASCAG-3′) and 806R (5′-GGACTACHNGGGTATCTAAT-3′). PCR amplification was carried out in a 30 μL reaction system containing 15 μL Phusion^®^ High-Fidelity PCR Master Mix (New England Biolabs, USA), 0.2 μmol/L of each forward and reverse primer, and approximately 10 ng of normalized template DNA. Subsequently, the constructed PCR libraries were sequenced on the Illumina HiSeq sequencing platform. Operational taxonomic units (OTUs) were clustered at a 97% sequence similarity cutoff value. For microbial diversity analysis, α-diversity indices were calculated from the normalized sequencing data, whereas β-diversity was assessed through principal component analysis (PCA).

### Cell culture and treatment

The Hepa 1-6 cell line was cultured in DMEM (Gibco, Missouri, USA) supplemented with 10% fetal bovine serum (FBS) (Gibco, Missouri, USA) and 1% penicillin–streptomycin liquid (Gibco, Missouri, USA). For BA treatment, when cells in 24-well plates exhibited robust growth and reached approximately 70% confluency, TLCA (Med Chem Express, cat. HY-113308A) was solubilized in the culture medium to achieve final concentrations of 0, 1, 5, 10, 20, and 50 μmol/L. Following 24 or 48 h of treatment, total RNA or total cellular protein was extracted for subsequent analysis. Based on previous studies, cells were treated with 20 μmol/L CDCA (Med Chem Express, cat. HY-76847) and 80 μmol/L benfluorex hydrochloride (Med Chem Express, cat. HY-B1058), which served as positive controls for FXR and HNF4α activation [31], respectively. In FXR and HNF4α inhibitor assays, cells were co-treated with 20 μmol/L TLCA and either 5 μmol/L DY268 (Med Chem Express, cat. HY-110267) or 2.5 μmol/L BI6015 (Med Chem Express, cat. HY-108469), respectively. All cell experiments were independently repeated at least three times.

### Real-time quantitative PCR

Total RNA was extracted from liver tissue using RNAiso Plus (TaKaRa, Code No. 9109). RNA concentration and purity were determined with a Nanodrop 2000 nucleic acid-protein analyzer (Thermo Fisher Scientific, USA). cDNA was synthesized using a reverse transcription kit (Vazyme Biotech Co., Ltd., Nanjing, China; catalog number R323-01). Quantitative PCR was performed with ChamQ Universal SYBR qPCR Master Mix (Vazyme Biotech Co., Ltd.; catalog number Q711) on an ABI QS5 Real-Time PCR System (Thermo Fisher Scientific, USA). Relative mRNA expression levels were calculated using the 2^−ΔΔCt^ method, with β-actin serving as the reference gene. Primers were synthesized by Sangon Biotech (Shanghai, China). The primer sequences for porcine tissues are listed in Table S2, and those for the murine Hepa1‑6 cell line are provided in Table S3.

### Western blotting analysis

Western blot analysis of hepatic proteins was performed according to established protocols [4]. Briefly, liver samples were pulverized in liquid nitrogen, and approximately 50 mg of powdered tissue was transferred to 2 mL microcentrifuge tubes containing ice-cold RIPA lysis buffer (P0013B, Beyotime Biotechnology, Shanghai, China). After thorough vortexing, samples underwent ultrasonic disruption for 5 min at 40 Hz under cryogenic conditions, followed by centrifugation at 12,000 × *g* for 30 min at 4 °C (Microfuge 20R, Beckman Coulter, USA). Supernatants were collected and mixed with 5 × SDS loading buffer supplemented with β-mercaptoethanol, then denatured by boiling at 95 °C for 10 min. Denatured proteins were separated by SDS-PAGE and transferred onto PVDF membranes. Membranes were blocked with 5% non-fat milk in TBST for 1 h at room temperature, followed by overnight incubation at 4 °C with primary antibodies diluted 1:1,000 in blocking buffer: rabbit anti-CYP27A1 (67045, Proteintech, USA), rabbit anti-SLC27A5 (84557, Proteintech, USA), rabbit anti-FXR/NR1H4 (72105, Cell Signaling Technology), and mouse anti-β-actin (20536-1-AP, Proteintech, USA). After washing three times with TBST (10 min each), the membranes were incubated with HRP-conjugated secondary antibodies (Beyotime Biotechnology, Shanghai, China) for 1 h at room temperature. Protein bands were visualized using an enhanced chemiluminescence (ECL) detection kit and imaged with a chemiluminescence imaging system (Tanon 5200, Shanghai, China). Densitometric analysis was performed using ImageJ software (NIH, USA), and target protein levels were normalized to β-actin.

### Molecular docking

Molecular docking was performed using AutoDock Tools and Auto Dock Vina (version 1.2.x), following established protocols [32], and the resulting complexes were visualized with PyMOL 2.6 software. Briefly, the 3D structures of HNF4α and FXR proteins were first obtained from the RCSB PDB database (https://www.rcsb.org) and preprocessed. Then, the 3D structure of TLCA (Compound CID: 439763) was obtained from the PubChem database (https://pubchem.ncbi.nlm.nih.gov/) and preprocessed. Finally, molecular docking was performed using AutoDock Tools with the appropriate docking mode and parameters.

### Statistical analysis

All data were analyzed using SAS software version 9.4 (SAS Institute Inc., Cary, NC, USA). The normality of data distributions was assessed using the Shapiro‒Wilk test. For comparisons between two groups, normally distributed data were analyzed using unpaired two-tailed Student’s *t*-tests, whereas non-normally distributed data were analyzed with the Wilcoxon rank-sum test. Comparisons among three or more groups were performed using one-way analysis of variance (ANOVA) followed by Dunnett’s multiple comparison test. Data are presented as means ± standard error of the mean (SEM). Correlation analyses were conducted only after verifying normality. Pearson correlation was applied when both datasets were normally distributed; otherwise, Spearman’s rank correlation was used. Spearman-based hierarchical clustering heatmaps with significance annotations were generated using OmicStudio (R version 3.6.3; https://www.omicstudio.cn). All graphical representations were prepared with GraphPad Prism version 9.0 (GraphPad Software, San Diego, CA, USA). Differences were considered statistically significant at *P* < 0.05, while 0.05 ≤ *P* < 0.10 was considered a tendency.

## Results

### LBW fetal pigs exhibited reduced hepatic glycogen storage and increased lipid accumulation

**Fig. 1:**
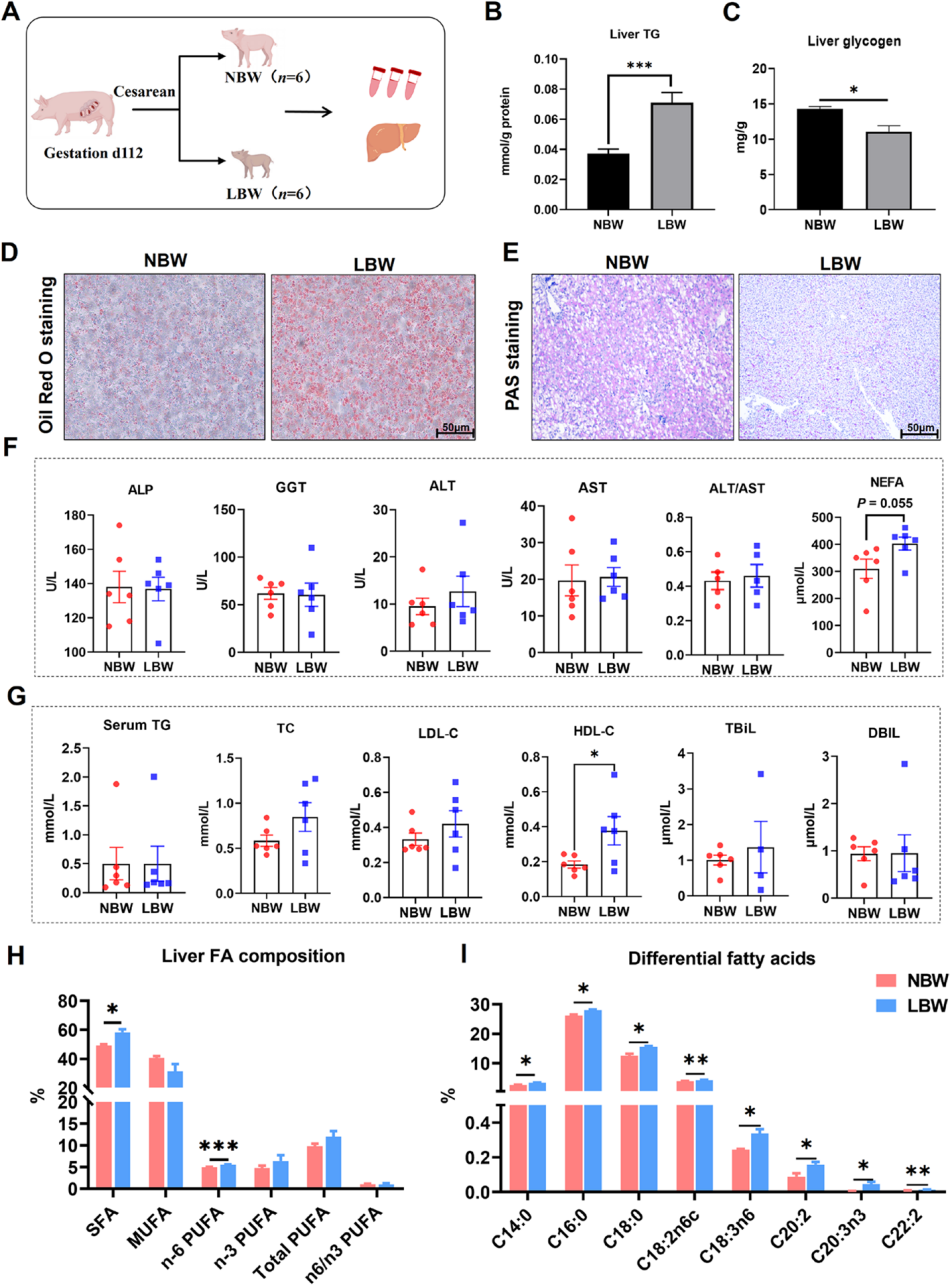
LBW fetuses exhibit reduced hepatic glycogen stores and increased hepatic lipid accumulation. **A** Experimental design. **B** Hepatic TG concentrations. **C** Hepatic glycogen concentrations. **D** Oil Red O staining of the liver tissue (200 ×, scale bar, 50 μm). **E** PAS staining of the liver tissue (200 ×, scale bar, 50 μm). **F** Serum ALP, GGT, ALT, AST activity, ratio of ALT/AST and the concentrations of NEFA in the serum. **G** TG, TC, HDL-C, LDL-C, TBiL, and DBiL concentrations in the serum. **H** and **I** Liver FA composition and differential fatty acids between the two groups. ^*^P < 0.05, ^**^P < 0.01, and ^***^P < 0.001, *n* = 6

The experimental design of the fetal pig study is shown in Fig. 1A. Hepatic triglyceride (TG) content was significantly increased (Fig. 1B, *P* < 0.001), whereas glycogen levels were markedly decreased (Fig. 1C, *P* < 0.05), in LBW fetal pigs compared with NBW controls, a finding further confirmed by Oil Red O staining and periodic acid–Schiff (PAS) staining (Fig. 1D and E). Since liver injury often alters serum biochemical parameters [33], a panel of markers was examined (Fig. 1F and G). Among these, serum HDL-C concentration was significantly higher in LBW fetal pigs than in NBW controls ((Fig. 1G, *P* < 0.05), whereas NEFA showed an increasing trend (Fig. 1F, *P* = 0.055). No significant differences were detected in ALP, GGT, ALT, AST, TG, TC, LDL-C, TBIL, DBIL, or the ALT/AST ratio between groups (Fig. 1F and G, *P* > 0.05).

Hepatic TG accumulation arises from an imbalance between fatty acid uptake, synthesis, and disposal [34]. To assess whether elevated TG levels were linked to changes in fatty acid composition, we analyzed hepatic free fatty acids (FFAs). The proportions of saturated fatty acids (SFA) (*P* < 0.05) and n-6 polyunsaturated fatty acids (n-6 PUFA) (*P* < 0.001) were significantly higher in LBW fetal pigs than in NBW controls (Fig. 1H). Further analysis revealed significant increases (*P* < 0.05) in C14:0 (myristic acid), C16:0 (palmitic acid), C18:0 (stearic acid), C18:2n6c (linoleic acid), C18:3n6 (γ-linolenic acid), C20:2 (eicosadienoic acid), C20:3n3 (eicosatrienoic acid), and C22:2 (docosadienoic acid) in LBW livers compared with NBW (Fig. 1I). Collectively, these results indicate that LBW fetal pigs exhibit excessive hepatic lipid deposition and impaired glycogen synthesis relative to NBW counterparts.

### Impaired HNF4α and FXR signaling underlies hepatic glycolipid metabolic dysfunction in LBW fetal pigs

**Fig. 2:**
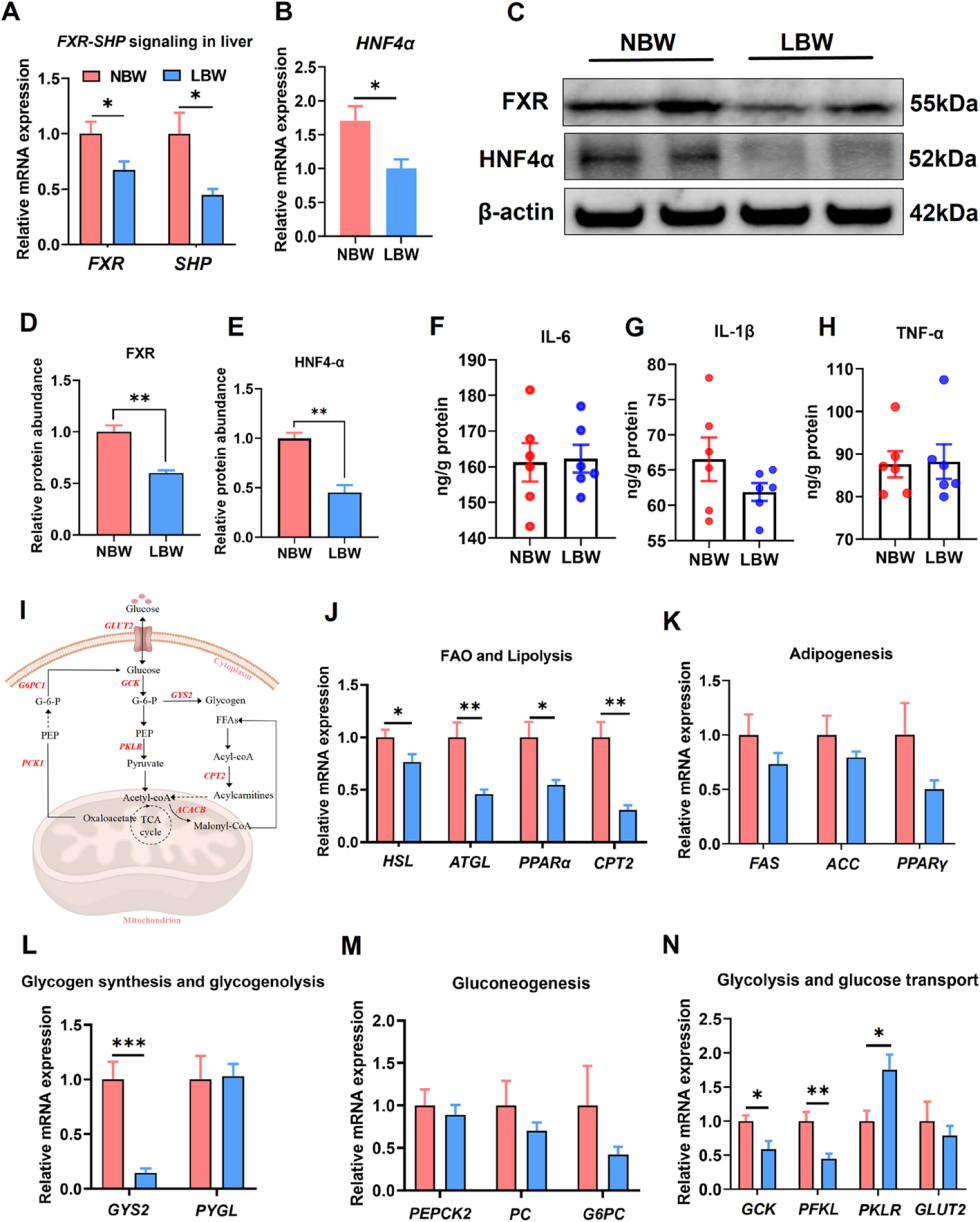
Impaired HNF4α and FXR signaling underlies hepatic glycolipid metabolic dysfunction in LBW fetal pigs. **A**–**E** Relative expression levels of FXR and HNF4α mRNA and proteins in the liver tissue. **F**–**H** Concentrations of IL-6, IL-1β, and TNF-α in the liver tissue. **I** Overview of hepatic fatty acid and glucose metabolic pathways. **J** and **K** Relative mRNA abundance of key genes involved in hepatic fatty acid metabolism. **L**–**N** Relative mRNA abundance of key genes involved in hepatic glycogen metabolism and transport. ^*^P < 0.05, ^**^P < 0.01, and ^***^P < 0.001, *n* = 6

HNF4α and FXR act as key regulators of hepatic glycogen and lipid metabolism [13, 35]. To investigate whether LBW-induced hepatic glycolipid dysregulation is associated with abnormal signaling responses of HNF4α and FXR, we determined the mRNA and protein expression levels of these two nuclear receptors in liver tissues. Compared with NBW fetal pigs, LBW fetal pigs exhibited significantly downregulated (*P* < 0.05) hepatic mRNA expression of *HNF4α*, *FXR*, and small heterodimer partner (*SHP*, target gene of FXR) (Fig. 2A and B). Further Western blot analysis confirmed that the protein levels of FXR and HNF4α were also significantly reduced (*P* < 0.01) in LBW livers (Fig. 2C–E). Given that hepatic lipid accumulation can induce inflammation [36], we measured hepatic levels of pro-inflammatory cytokines. However, no significant differences in IL-6, TNF-α, or IL-1β levels were observed between groups (Fig. 2F–H). We then assessed the expression of key genes in glycogen and lipid metabolism under the regulation of HNF4α and FXR (Fig. 2I). Genes involved in fatty acid β-oxidation, including hormone-sensitive lipase (*HSL*), adipose triglyceride lipase (*ATGL*), peroxisome proliferator-activated receptor alpha (*PPARα*), and carnitine palmitoyltransferase 2 (*CPT2*), were significantly downregulated (*P* < 0.05) in LBW livers compared with NBW (Fig. 2J). However, the expression of lipogenic genes (Fig. 2K), including fatty acid synthase (*FAS*), acetyl-CoA carboxylase (*ACC*), and peroxisome proliferator-activated receptor gamma (*PPARγ*), showed no significant differences between groups. These findings suggest that the significant downregulation of genes involved in fatty acid oxidation may contribute to hepatic lipid accumulation in LBW pigs, whereas dysregulated lipid synthesis is less likely to be the dominant cause. In terms of glucose metabolism, glycogen synthase 2 (*GYS2*) expression was markedly decreased (*P* < 0.001) in LBW livers compared with NBW controls, whereas glycogen phosphorylase (*PYGL*) expression did not differ significantly between the groups (Fig. 2L). The expression of gluconeogenic genes, including phosphoenolpyruvate carboxykinase 2 (*PEPCK2*), phosphatidyl choline (*PC*), and glucose-6-phosphatase catalytic subunit 1 (*G6PC*), was also comparable between groups (Fig. 2M). Notably, compared with NBW controls, LBW livers exhibited significantly downregulated (*P* < 0.05) expression of glucokinase (*GCK*) and liver-type phosphofructokinase (*PFKL*) (Fig. 2N), whereas pyruvate kinase L/R (*PKLR*) was upregulated (*P* < 0.05). No significant differences were observed in the expression of glucose transporter 2 (*GLUT2*) between LBW and NBW livers. In summary, impaired HNF4α and FXR signaling, together with reduced expression of their target genes governing glycogen synthesis and fatty acid oxidation, contributes to hepatic glycolipid metabolic dysfunction in LBW fetal pigs.

### LBW fetal pigs exhibit altered hepatic BA composition and reduced TLCA levels

**Fig. 3:**
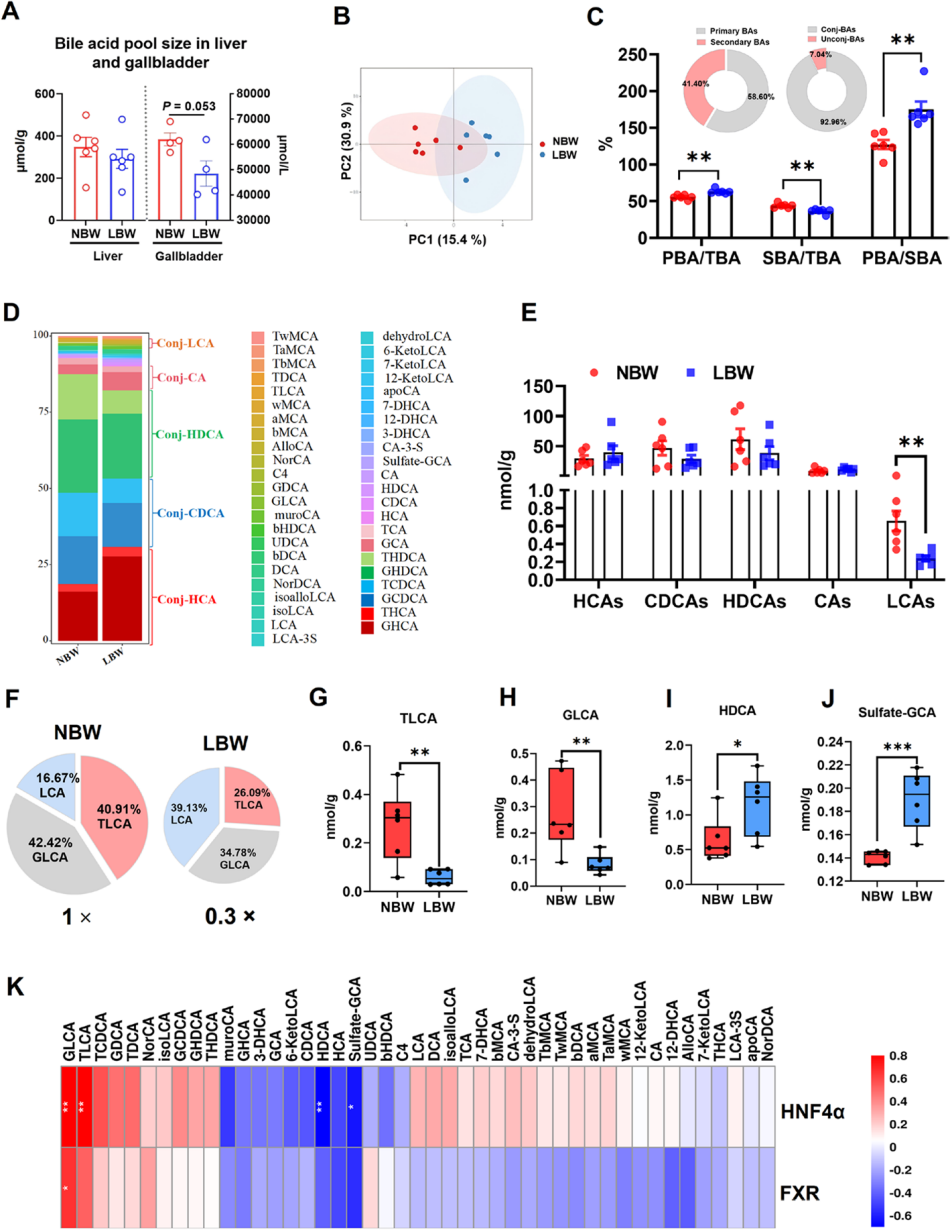
LBW fetal pigs exhibit altered hepatic BA composition and reduced levels of TLCA. **A** Total BA content of liver and gallbladder contents. **B** PCA of BAs composition in the liver. **C** Overview of the proportions of primary or secondary BAs, as well as conjugated or unconjugated. The ratio of primary BAs and secondary BAs to total BAs, and the ratio of primary BAs to secondary BAs. **D** and **E** Classification and differential analysis of major BA composition in the liver of fetal pigs. **F** Proportions of LCA, TLCA, and GLCA to LCAs. **G**–**J** Differential BAs between the two groups. **K** Correlation between the relative expression abundance of hepatic *HNF4α* and *FXR* genes and BA concentrations. ^*^P < 0.05, ^**^P < 0.01, and ^***^P < 0.001, *n* = 6

BAs act as endogenous ligands for multiple hepatic nuclear receptors, modulating their conformation and transcriptional activity [1]. As shown in Fig. 3A, no significant differences were observed in the sizes of the BA pools in the liver and gallbladder between LBW and NBW fetal pigs. To determine whether impaired HNF4α and FXR signaling in LBW fetal pig livers is associated with altered BA composition, we profiled hepatic BAs using targeted metabolomics. In total, 45 BA species were detected (Table S4). Principal component analysis (PCA) revealed a clear separation of BA composition between LBW and NBW groups (Fig. 3B). Overall, primary BAs accounted for 58.6% of total hepatic BAs in fetal pigs, while 92.96% of all BAs were present in conjugated forms (Fig. 3C). Although absolute levels of primary BAs (PBA), secondary BAs (SBA), and total BAs (TBA) did not differ between groups (Fig. S1, *P* > 0.05), LBW livers exhibited a significantly higher proportion of PBA (*P* < 0.01) and a lower proportion of SBA (*P* < 0.01) compared with NBW, resulting in a significantly increased PBA/SBA ratio (Fig. 3C, *P* < 0.01). These findings indicate disrupted hepatic BA homeostasis in LBW fetal pigs. Hyocholic acid (HCA), CDCA, hyodeoxycholic acid (HDCA), cholic acid (CA), and lithocholic acid (LCA) are the predominant BA species in porcine systemic circulation [37]. In fetal pig livers, more than 85% of total BAs were composed of these major species and their conjugated forms (Fig. 3D). Notably, compared with NBW piglets, only lithocholic acid derivatives (TLCA + GLCA + LCA) were significantly reduced in LBW livers (Fig. 3E, *P* < 0.01), with TLCA exhibiting the most pronounced decline, decreasing from 40.91% in NBW to 26.09% in LBW (Fig. 3F).

Further analysis revealed that four BAs differed significantly between NBW and LBW livers. TLCA exhibited the largest change, with levels in LBW livers reduced by approximately 79% relative to NBW (Fig. 3G, *P* < 0.01). GLCA was similarly decreased by about 71% (Fig. 3H, *P* < 0.01). In contrast, compared with NBW piglets, HDCA and GCA were elevated in LBW livers, increasing by 1.8-fold (Fig. 3I, *P* < 0.05) and 1.4-fold (Fig. 3J, *P* < 0.001), respectively. Spearman correlation analysis revealed that TLCA levels were positively associated with hepatic HNF4α (*P* < 0.01) and showed a strong correlation with FXR expression (Fig. 3K). Overall, these findings suggest that diminished TLCA abundance in LBW livers may contribute to impaired HNF4α and FXR signaling.

### LBW alters hepatic BA metabolism and impairs the biosynthesis of TLCA

**Fig. 4:**
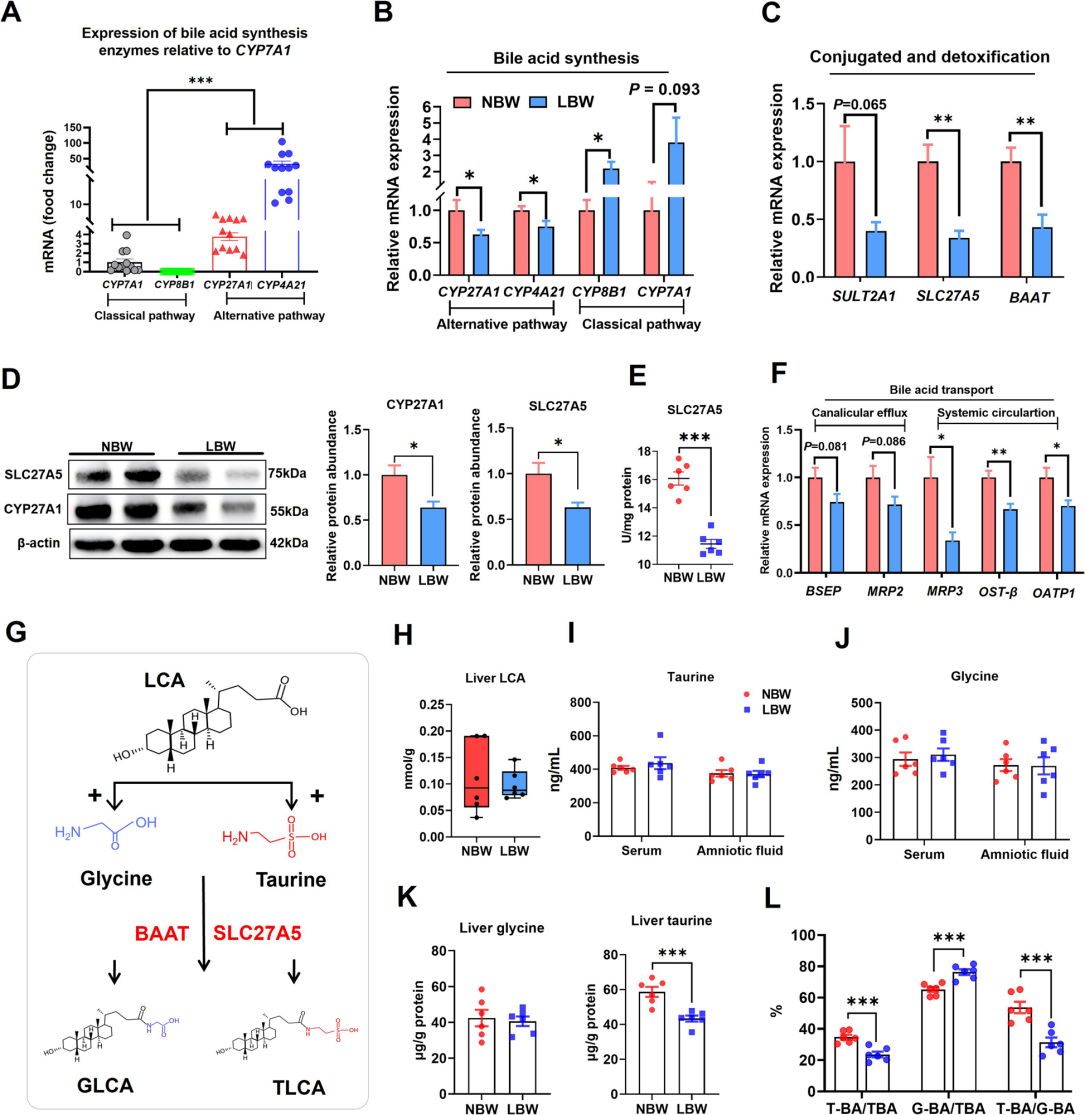
LBW alters hepatic BA metabolism and impairs the biosynthesis of TLCA in fetal pigs. **A** and **B** Relative mRNA abundance of genes involved in regulating BA synthesis in the liver. **C** Relative mRNA abundance of genes involved in regulating BA detoxification and conjugation in the liver. **D** Protein expression levels of SLC27A5 and CYP27A1 in the liver. **E** Detection of SLC27A5 protein expression in the liver by ELISA. **F** Relative mRNA abundance of genes involved in regulating BA transport in the liver. **G** Biosynthetic process of TLCA and GLCA in the liver. **H** LCA concentrations in the liver. **I**–**J** Glycine and taurine concentrations in serum and amniotic fluid. **K** Glycine and taurine concentrations in liver. **L** The ratio of taurine-conjugated BAs and glycine-conjugated BAs in the liver. ^*^P < 0.05, ^**^P < 0.01, and ^***^P < 0.001, *n* = 6

To elucidate the mechanisms responsible for altered BA composition and TLCA deficiency, we examined key genes and proteins involved in BA synthesis, conjugation, transport, and detoxification. In adults, the classical BA synthesis pathway, primarily mediated by cholesterol 7α-hydroxylase (CYP7A1), accounts for over 90% of total BA production [1]. In fetal livers, however, the alternative pathway, initiated by cholesterol 27-hydroxylase (*CYP27A1*), predominated (Fig. 4A, *P* < 0.001). Notably, compared to NBW, the alternative pathway was significantly downregulated in LBW livers, whereas the classical pathway was upregulated (Fig. 4B, *P* < 0.05). Before being secreted into the gallbladder, BAs undergo hepatic conjugation or sulfation [2]. Analysis of related genes and proteins showed that sulfotransferase family 2 A member 1 (SULT2A1), responsible for sulfation, tended to be lower in LBW livers without statistical significance (Fig. 4C, *P* = 0.065). Consistent with this, SULT2A1 protein levels in serum and liver and sulfated BA levels were unchanged across groups (Fig. S1, *P* > 0.05). More critically, compared with NBW, both solute carrier family 27 member 5 (*SLC27A5*) and bile acid-CoA:amino acid N-acyltransferase (*BAAT*) mRNA levels, encoding key enzymes for BA conjugation [38], were markedly reduced in LBW livers (Fig. 4C, *P* < 0.01). In line with this, SLC27A5 protein abundance and its enzymatic activity were also significantly decreased in the LBW group (Fig. 4D and E, *P* < 0.05). Furthermore, compared to NBW livers, genes involved in BA transport also exhibited significantly decreased expression in LBW livers, indicating impaired hepatic BA transport capacity (Fig. 4F,* P* < 0.05).

The biosynthesis of TLCA involves LCA as the substrate for conjugation with taurine or glycine, catalyzed by *SLC27A5* and *BAAT* to form TLCA or GLCA (Fig. 4G) [39]. As LCA levels did not differ significantly between groups (Fig. 4H, *P* > 0.05), we further quantified glycine and taurine levels in fetal serum, amniotic fluid, and liver tissue. Glycine levels showed no significant differences among LBW and NBW groups in serum, amniotic fluid, or liver (Fig. 4I–K). In contrast, taurine concentrations were specifically reduced in LBW livers (Fig. 4K, *P* < 0.001), while serum and amniotic fluid levels remained comparable between groups (Fig. 4I). Consequently, the proportion of taurine-conjugated BAs relative to total BAs was lower in LBW livers (Fig. 4L, *P* < 0.001).

**Fig. 5:**
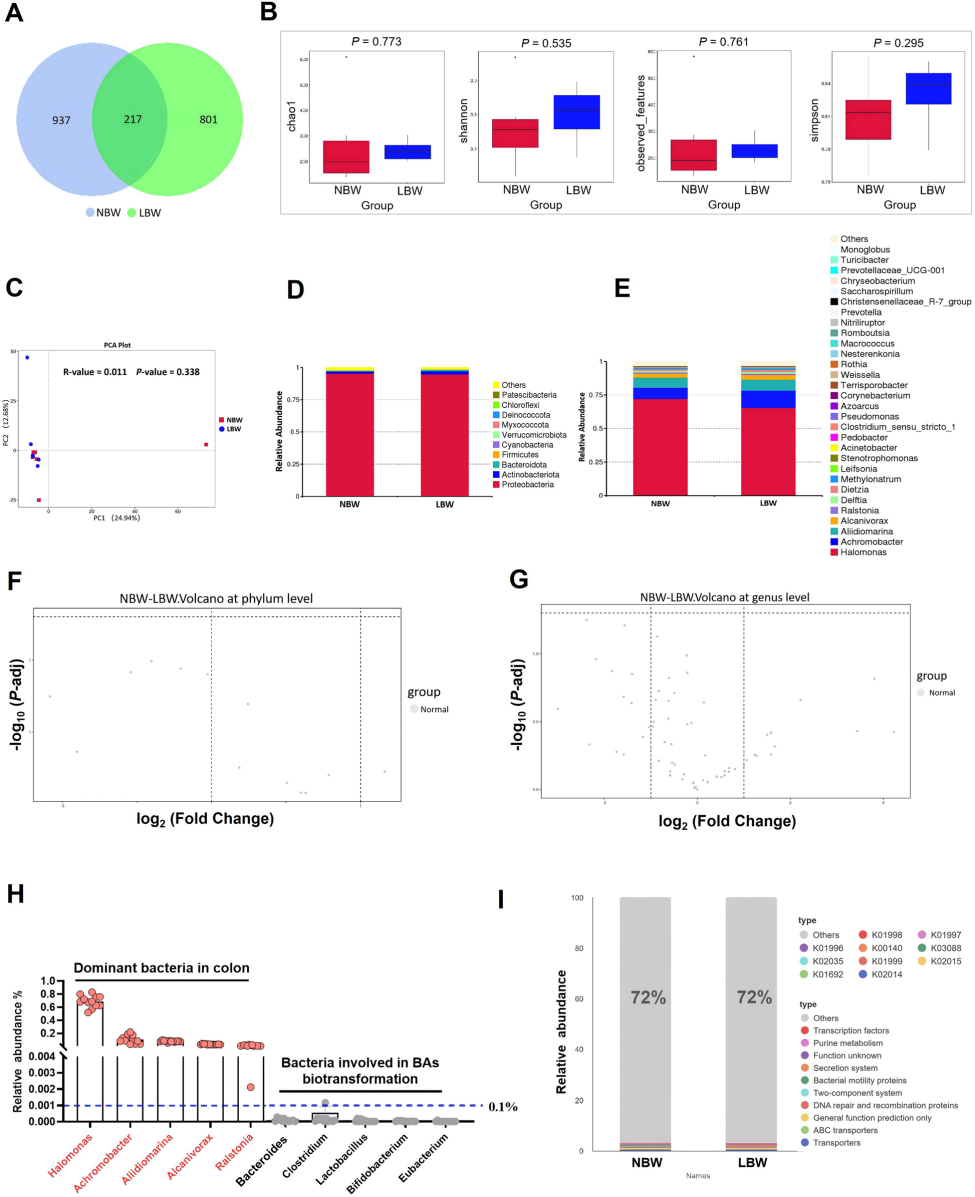
16S rRNA gene sequencing analysis of cecal microbial community characteristics in NBW and LBW fetal pigs. **A** Venn diagram showing the distribution of operational taxonomic units (OTUs). **B** Analysis of α-diversity indices of cecal microbial communities. **C** Principal component analysis (PCA) of cecal microbial community structure. **D** and **E** Relative abundance of dominant microbial taxa at the phylum and genus levels. **F** and **G** Volcano plots of differential microbial taxa between NBW and LBW groups at the phylum and genus levels. **H** Relative abundance of core microbial genera and BA metabolism-related genera. **I** Functional prediction of cecal microbial communities. *n* = 6

It is well established that gut microbes participate in the synthesis and biotransformation of secondary BAs. To further explore whether fetal BA metabolism is associated with the gut microbiota, we performed microbial diversity sequencing analysis on colonic contents of fetal pigs. Our results demonstrated no significant differences in α-diversity and β-diversity of colonic microbiota between NBW and LBW fetal pigs (Fig. 5A–C). At the phylum level, the dominant taxa were Proteobacteria and Actinobacteriota, which collectively accounted for more than 90% of the total microbial community (Fig. 5D). These two phyla are predominantly distributed in soil or contaminated environments, rather than serving as the dominant commensals in the porcine gut. Similarly, at the genus level, the dominant genera were *Halomonas*, *Achromobacter*, and *Aliidiomarina*, all of which belong to the phylum Proteobacteria. These genera were also mainly distributed in soil or environmental niches instead of being the core gut microbiota of pigs, and collectively constituted approximately 90% of the total microbial community (Fig. 5E). Volcano plot analysis revealed no differentially abundant microbes at either the phylum or genus level between the two groups (Fig. 5F and G). We further quantified the abundance of bacterial taxa involved in BA synthesis and biotransformation in the gut, and their relative abundance was found to be below 0.1% (Fig. 5H). In addition, bacterial functional prediction showed an extremely low proportion of microbial functions enriched in core vital activities in both groups (Fig. 5I). Collectively, these findings indicate that LBW fetal pigs exhibit disrupted hepatic BA metabolism and TLCA deficiency, which is unrelated to fetal gut microbial activity and primarily attributable to impaired taurine availability and reduced expression of SLC27A5 and BAAT. These alterations likely contribute to the attenuation of HNF4α and FXR signaling and downstream metabolic dysregulation.

### Supplementation of TLCA ameliorates hepatic glycolipid dysregulation in neonatal LBW pigs

**Fig. 6:**
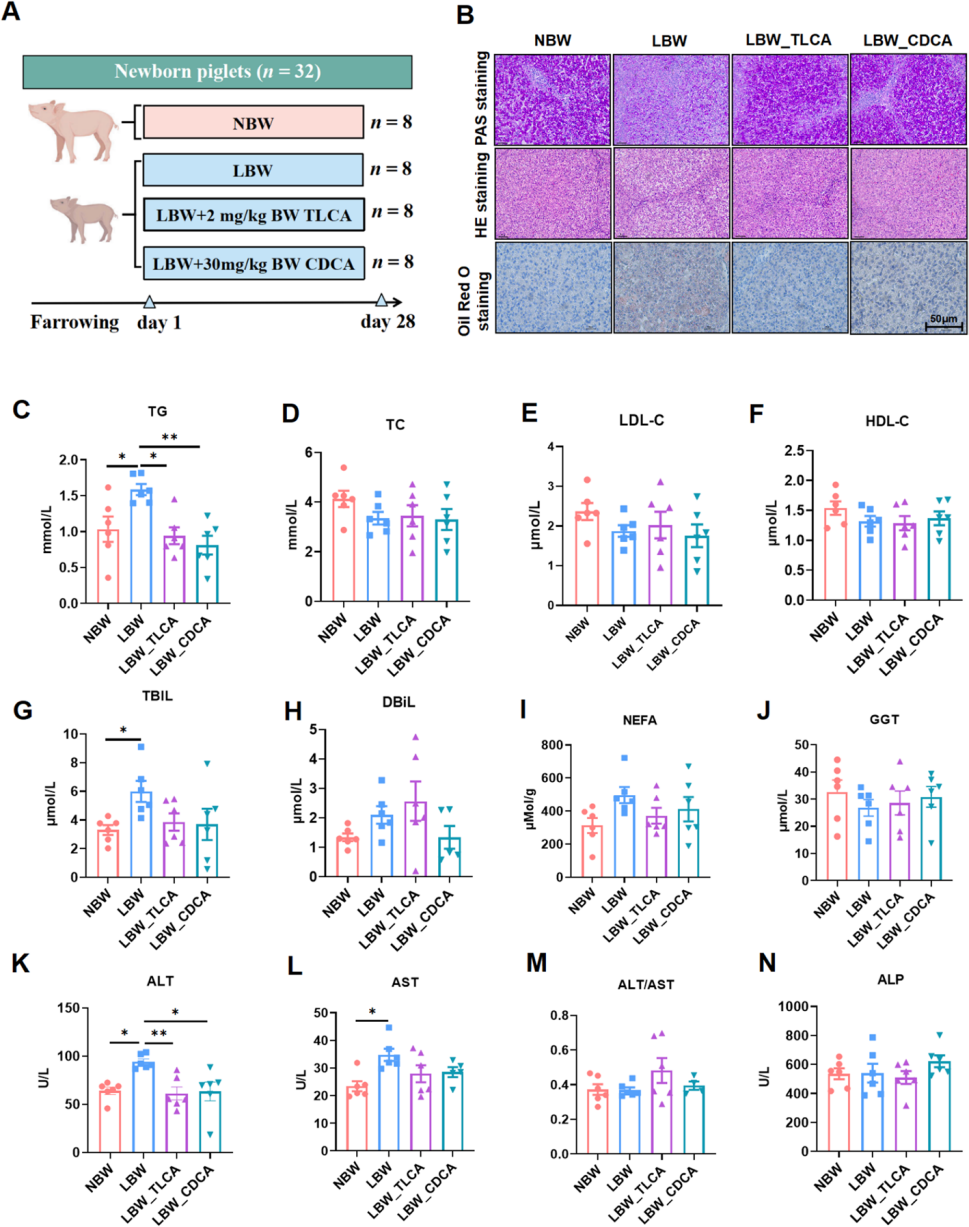
Supplementation of TLCA ameliorates hepatic glucose-lipid dysregulation in newborn LBW piglets. **A** Experimental design. **B** PAS staining, H&E staining and Oil Red O staining of the liver tissue (200 ×, scale bar, 50 μm). **C**–**N** Serum TG, TC, LDL-C, HDL-C, TBIL, DBIL, and NEFA concentrations, serum ALP, GGT, ALT, and AST activity and the ratio of ALT/AST. ^*^P < 0.05, ^**^P < 0.01, and ^***^P < 0.001, *n* = 6

To validate the critical role of TLCA in hepatic glycolipid metabolism, in vivo experiments were performed in LBW pigs (Fig. 6A). CDCA, the most potent natural endogenous FXR agonist [40], was used as a positive control. Consistent with observations in LBW fetal pigs, hepatic glycogen content in LBW piglets was lower than that in NBW controls (Fig. 6B). Supplementation with TLCA or CDCA effectively restored glycogen levels to values comparable with those in NBW piglets. H&E and Oil Red O staining revealed pronounced lipid droplet deposition in the livers of LBW compared with NBW piglets. This pathological accumulation was markedly attenuated by TLCA or CDCA supplementation (Fig. 6B). Serum biochemical analysis further corroborated these findings. Compared to NBW piglets, LBW piglets exhibited significantly elevated levels of TG, TBIL, ALT, and AST (Fig. 6C–N, *P* < 0.05). Supplementation with TLCA or CDCA partially or fully ameliorated these alterations, reflecting improved liver function. In summary, these results demonstrate that TLCA and CDCA supplementation effectively mitigate hepatic glycolipid dysregulation and enhances liver health in newborn LBW piglets.

### TLCA regulates hepatic glycolipid metabolism by activating FXR and HNF4α in vitro and in vivo

**Fig. 7:**
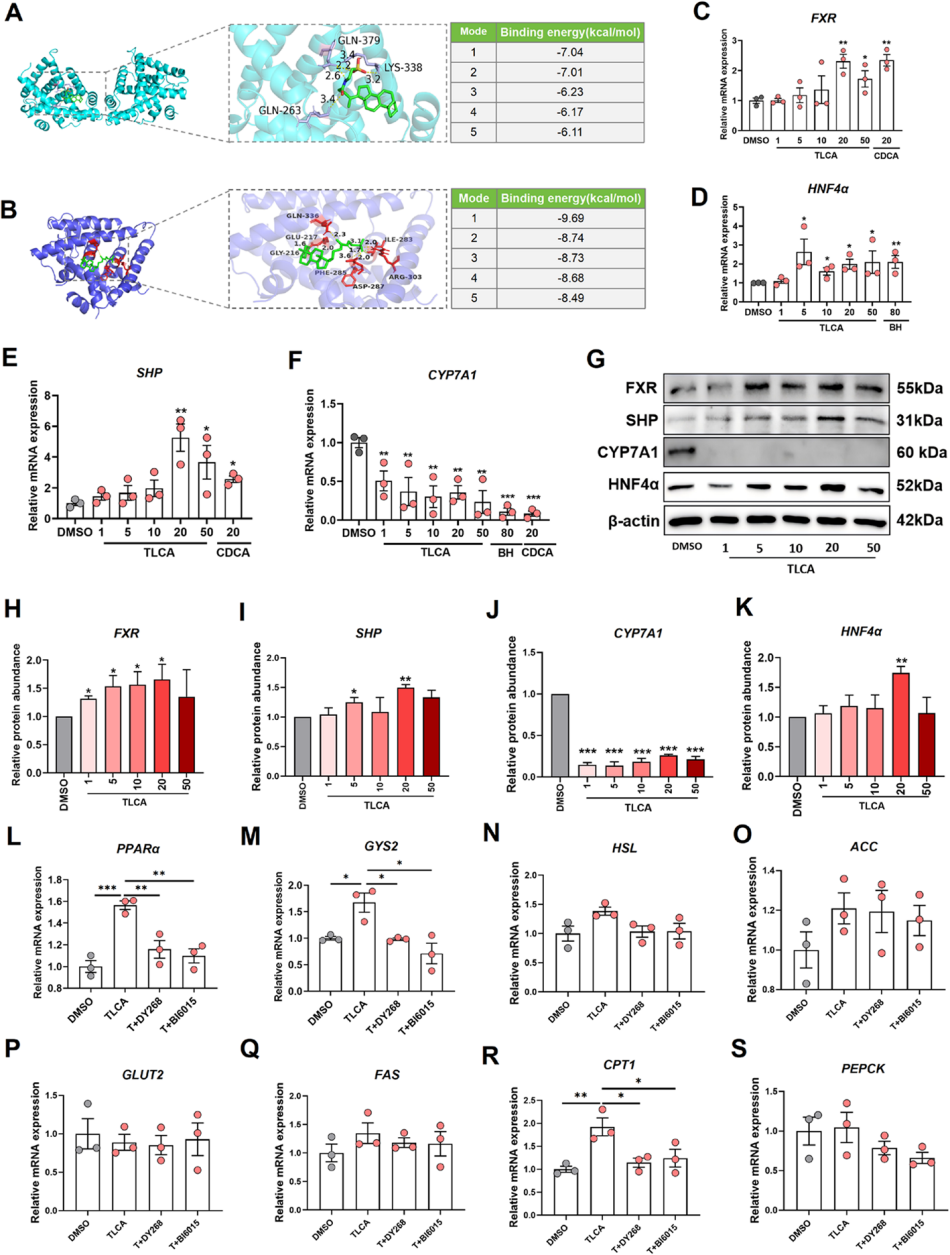
TLCA regulates hepatic glycolipid metabolism via HNF4α and FXR signaling in Hepa 1-6 cells. **A** and **B** 3D structures of TLCA-FXR and TLCA-HNF4α molecular docking complexes. **C**–**F** FXR/HNF4α signaling-related gene expression in Hepa 1-6 cells following treatments with 0, 1, 5, 10, 20 and 50 μmol/L TLCA for 24 h. CDCA (FXR agonist, 20 μmol/L) and BH (HNF4α agonist, 80 μmol/L) were used as positive controls. **G**–**K** Protein expression of FXR, HNF4α, SHP and CYP7A1 in Hepa1-6 cells treated with TLCA for 48 h, with β-actin as the loading control. **L**–**S** Expression of glycolipid metabolism-related genes after 20 μmol/L TLCA treatment for 24 h. DY268 (5 μmol/L) and BI6015 (2.5 μmol/L) are competitive inhibitors of FXR and HNF4α, respectively. TLCA, taurolithocholic acid; CDCA, chenodeoxycholic acid; BH, benfluorex hydrochloride. ^*^P < 0.05, ^**^P < 0.01, and ^***^P < 0.001, *n* = 3

To elucidate the mechanism by which TLCA alleviates liver glucose and lipid metabolism disorders in LBW piglets, molecular docking simulations demonstrated that TLCA has a strong binding affinity for the nuclear proteins FXR and HNF4α, with minimum binding energies of −7.04 and −9.69 kcal/mol, respectively (Fig. 7A and B). To verify the activating effect of TLCA on FXR and HNF4α, studies were performed in Hepa 1-6 cells. TLCA activated the mRNA and protein expression of FXR and HNF4α in a dose-dependent manner, with the most pronounced effect observed at a concentration of 20 μmol/L (Fig. 7C–K, *P* < 0.05). The positive responses of their downstream target genes, *SHP* and cytochrome P450 family 7 subfamily A member 1 (*CYP7A1*), confirmed the simultaneous activation of FXR and HNF4α by TLCA (Fig. 7E, F, I, and J; *P* < 0.01). Subsequently, we further validated whether the regulatory effect of TLCA on hepatic glycolipid metabolism is dependent on FXR and HNF4α (Fig. 7L–S). The results demonstrated that, compared with the control group, TLCA treatment significantly upregulated (*P* < 0.05) the mRNA expression of *PPARα*, glycogen synthase 2 (*GYS2*), and carnitine palmitoyltransferase 1 (*CPT1*). However, this promotional effect was significantly abrogated when TLCA was co-treated with either FXR or HNF4α inhibitor. Collectively, these findings confirm that TLCA regulates hepatic glycolipid metabolism homeostasis in an FXR- and HNF4α-dependent manner.

**Fig. 8:**
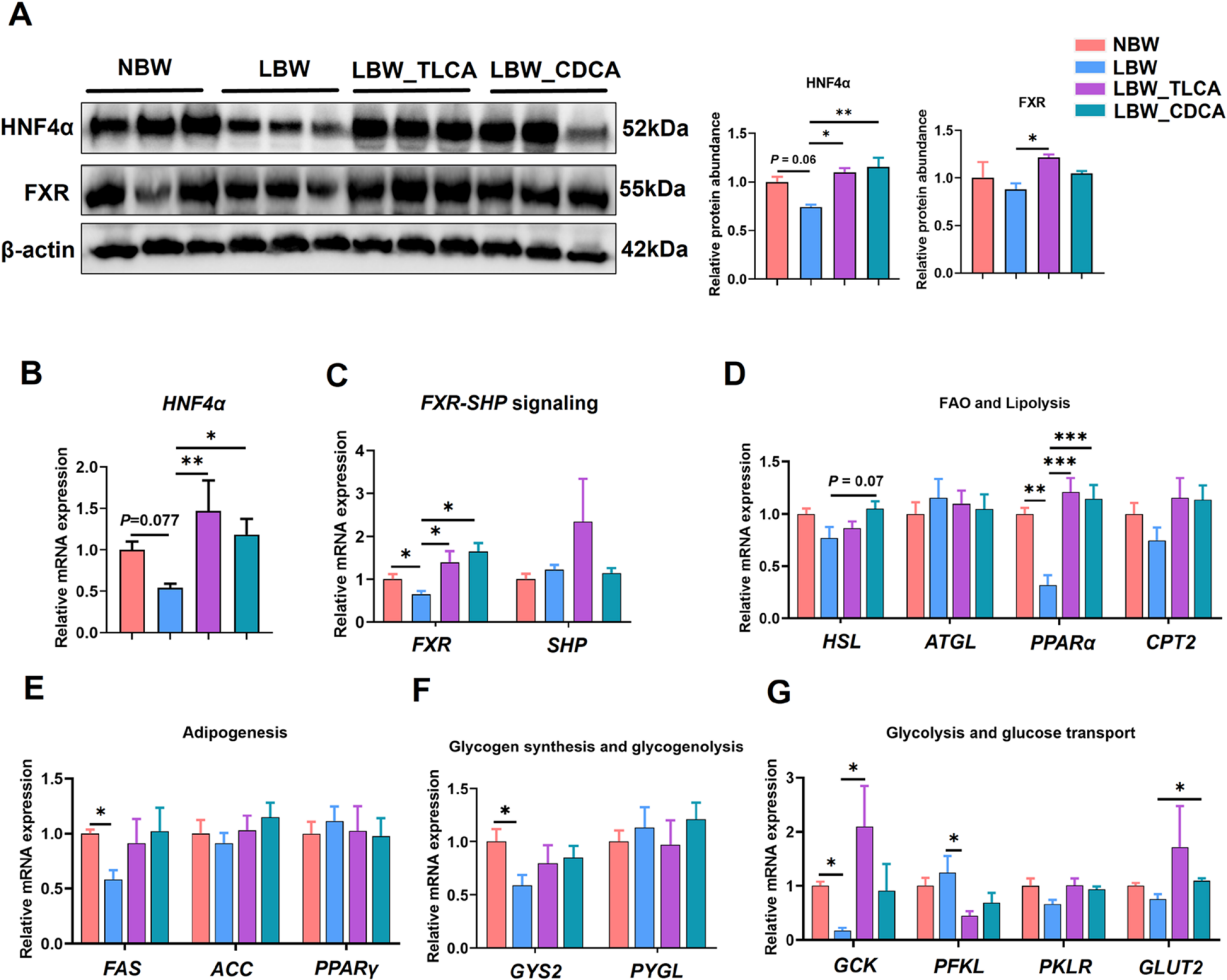
TLCA modulates hepatic glycolipid metabolism through HNF4α and FXR. **A** Relative protein expression levels of FXR and HNF4α in the liver. **B** and **C** Relative mRNA abundance of *HNF4α* and *FXR* target genes in the liver. **D** and **E** Relative mRNA abundance of key genes involved in hepatic fatty acid metabolism. **F** and **G** Relative mRNA abundance of key genes involved in hepatic glycogen metabolism and glucose transport. ^*^P < 0.05, ^**^P < 0.01, and ^***^P < 0.001, *n* = 6

Subsequently, we further verified whether TLCA exerts its biological function in vivo. Protein analysis revealed distinct patterns for HNF4α and FXR (Fig. 8A). Hepatic HNF4α protein abundance was reduced (*P* = 0.06) in LBW piglets compared with NBW, and supplementation with TLCA or CDCA restored its expression to levels comparable with NBW (*P* < 0.05). In contrast, FXR protein levels did not differ significantly between LBW and NBW controls. However, TLCA supplementation markedly increased FXR protein expression relative to LBW (*P* < 0.05), whereas CDCA treatment showed no significant effect on FXR protein expression. Consistent with the protein results, both *FXR* and *HNF4α* mRNA levels were reduced in the liver of LBW piglets compared with NBW piglets, whereas supplementation with TLCA or CDCA significantly upregulated their expression (Fig. 8B and C, *P* < 0.05). Furthermore, compared with NBW piglets, the relative expression level of hepatic *PPARα* (Fig. 8D), a key gene involved in fatty acid oxidation, was significantly reduced in LBW piglets, whereas supplementation with TLCA or CDCA markedly reversed this reduction (*P* < 0.01). *FAS* expression was significantly reduced (*P* < 0.05) in LBW piglets compared with NBW, whereas TLCA or CDCA supplementation abrogated this reduction (Fig. 8E). *ACC* and *PPARγ* showed no significant variation across the four groups. In glycogen metabolism, *GYS2* expression was significantly lower (*P* < 0.05) in LBW piglets than in NBW, but was restored by TLCA or CDCA supplementation (Fig. 8F). However, *PYGL* expression showed no significant differences among the groups. Furthermore, the expression level of GCK in LBW piglets was significantly lower (*P* < 0.05) than that in NBW piglets, and supplementation with TLCA significantly increased *GCK* expression compared with the LBW group, while no similar effect was observed following CDCA supplementation (Fig. 8G). In contrast, compared with the LBW group, TLCA supplementation significantly decreased (*P* < 0.05) the expression level of *PFKL*, whereas no such effect was noted in the CDCA-supplemented group (Fig. 8G). Interestingly, the expression of GLUT2 was significantly higher (*P* < 0.05) in the CDCA group than in the LBW group, and no differences were observed among the other groups (Fig. 8G). Overall, these findings indicate that TLCA modulates hepatic glycolipid metabolism in LBW piglets primarily through activation of the HNF4α and FXR signaling.

## Discussion

Existing studies have confirmed that LBW is associated with metabolic syndrome in humans and animal models, including obesity, type 2 diabetes, cardiovascular diseases, and liver diseases [41]. Consistent with the observations in FGR phenotypes in humans and mice [41, 42], the present study found that LBW in fetal and newborn piglets leads to significant abnormalities in hepatic glucose and lipid metabolism, which are typically characterized by intrahepatic lipid deposition and inhibition of hepatic glycogen synthesis. Notably, the hepatic lipid deposition in LBW fetal pigs is mainly caused by reduced fatty acid oxidation rather than enhanced lipid synthesis. This is in line with the findings of Liu et al. in weaned IUGR piglets [43]. Similarly, the deficiency of hepatic glycogen results from the inhibition of glycogen synthesis capacity mediated by *GYS2*, rather than increased glycogen consumption. On the contrary, the expression of genes involved in glycolysis is downregulated in the liver of LBW fetal pigs. Importantly, these abnormalities in the liver of LBW fetal pigs persist into the neonatal period, indicating the existence of hepatic persistent metabolic programming. This is consistent with the view proposed in the "fetal origins hypothesis" by David Barker and colleagues, which states that "intrauterine growth restriction increases susceptibility to metabolic diseases in adulthood" [44].

Abnormal methylation of specific HNF4α promoters may be one of the mechanisms by which IUGR increases susceptibility to long-term diseases, such as type 2 diabetes mellitus (T2DM) [45]. However, there are very few studies on the expression pattern of hepatic HNF4α in LBW fetal pigs. Studies in rats have shown that, compared with NBW rats, there is no significant change in hepatic HNF4α in LBW rats [46]. However, our research on the pig model revealed that the expression of HNF4α in the liver of LBW fetal pigs and newborn piglets is significantly lower than that in NBW counterparts, suggesting that the deficiency of HNF4α may be a key factor leading to hepatic glucose and lipid metabolism disorders in LBW pigs. This result indicates that, compared with rodent models, the pig model may have greater and unique reference value in the research of human diseases. Another key protein that regulates hepatic glucose and lipid metabolism is FXR [17]. A large number of studies have demonstrated that FXR not only plays an important role in BA metabolism but also acts as a key regulator in the maintenance of lipid and glucose homeostasis [47–49]. However, to our knowledge, the expression pattern of hepatic FXR in LBW fetal pigs remains unclear. In our study, we found that mRNA and protein expressions of FXR in the liver of LBW pigs were significantly lower than those in NBW pigs. Moreover, PPARα is a common downstream target of both HNF4α and FXR [50, 51], playing a central role in regulating hepatic fatty acid β-oxidation and lipid metabolism. Consistent with this regulatory axis, our study demonstrated that hepatic PPARα expression was markedly reduced in LBW fetal and neonatal pigs, whereas supplementation with TLCA or CDCA restored its expression, thereby contributing to the improvement of hepatic lipid metabolism.

Previous studies have reported changes in BA composition in newborn or weaned LBW piglets [21]. However, it remains unclear whether the BA composition in the liver of LBW fetal pigs is altered. Our results indicate that although the total BA pool remains unchanged, the increased proportion of primary BAs and decreased proportion of taurine-conjugated BAs in the liver of LBW fetal pigs fully demonstrate abnormal BA metabolism in these animals. Further verification at the gene level confirms the presence of disorders in hepatic BA metabolism in LBW fetal pigs. Furthermore, we found that BA synthesis during the fetal period is dominated by the alternative synthetic pathway, which is consistent with the findings of Vonderohe et al. [52]. More importantly, the present study found that endogenous TLCA is depleted in the liver of LBW fetal pigs. SLC27A5 and BAAT are key enzymes responsible for the synthesis of conjugated BAs in the liver [39]. Recent studies have revealed that deletion of hepatic SLC27A5 in mice leads to liver injury [53]. Our study showed that mRNA and protein expression levels of SLC27A5 in the liver of LBW fetal pigs are significantly lower than those in NBW fetal pigs. These results suggest that the downregulation of SLC27A5 and BAAT is a contributing factor to TLCA depletion. Taurine and glycine are substrates for conjugated BAs, which are synthesized by SLC27A5 and BAAT enzymes [1]. However, it has not yet been reported whether the contents of taurine and glycine in the liver of LBW fetal pigs are deficient. Our study found a specific deficiency of taurine in the liver of LBW fetal pigs, while taurine levels in serum and amniotic fluid remained unchanged. These results suggest that taurine deficiency may be another significant factor contributing to the reduction in TLCA synthesis. TLCA is a secondary BA whose synthesis is dependent on the intestinal microbiota. However, since the fetal intestine is considered sterile [54], fetuses lack the capacity to produce TLCA on their own. Our previous study has demonstrated that maternal–fetal BA transfer, including that of LCA, occurs during gestation (on d 60, d 90, and the day of parturition) [55]. More importantly, in another unpublished study, we confirmed that TLCA was undetectable in placental tissues of fetal pigs. This finding indicates that TLCA is not directly transferred from the mother to the fetus, but rather endogenously synthesized in the fetal liver using maternally derived LCA as the substrate.

Studies have demonstrated that FXR and HNF4α can undergo co-immunoprecipitation in the mouse liver [56]. Further analysis using chromatin immunoprecipitation sequencing (ChIP-seq) revealed that nearly 50% of FXR and HNF4α binding sites in the mouse liver overlap [56]. More importantly, transcriptional and binding assays indicated that HNF4α can enhance the transcriptional activity of FXR [56]. These findings suggest that, in our study, TLCA may initially activate HNF4α, thereby subsequently increasing FXR transcription. Although the molecular mechanism by which TLCA activates HNF4α remains unclear, in vitro studies have confirmed that the secondary BA DCA can activate HNF4α protein expression in a dose-dependent manner [22], indicating that BAs can directly regulate HNF4α expression patterns. In the present study, we found that 20 μmol/L TLCA could activate both FXR and HNF4α in Hepa 1-6 cells. Although our study has confirmed the ameliorative effect of TLCA on hepatic metabolic disorders in LBW piglets, several limitations remain. In our current research, the impact of gender on the study results has not been considered, which is a crucial factor, as accumulating evidence has indicated that liver injury in IUGR fetuses is associated with gender [8, 57]. Furthermore, owing to the limited available sample size, the study was unable to perform sample collection with a paired design. These limitations will be addressed in future studies.

## Conclusion

**Fig. 9:**
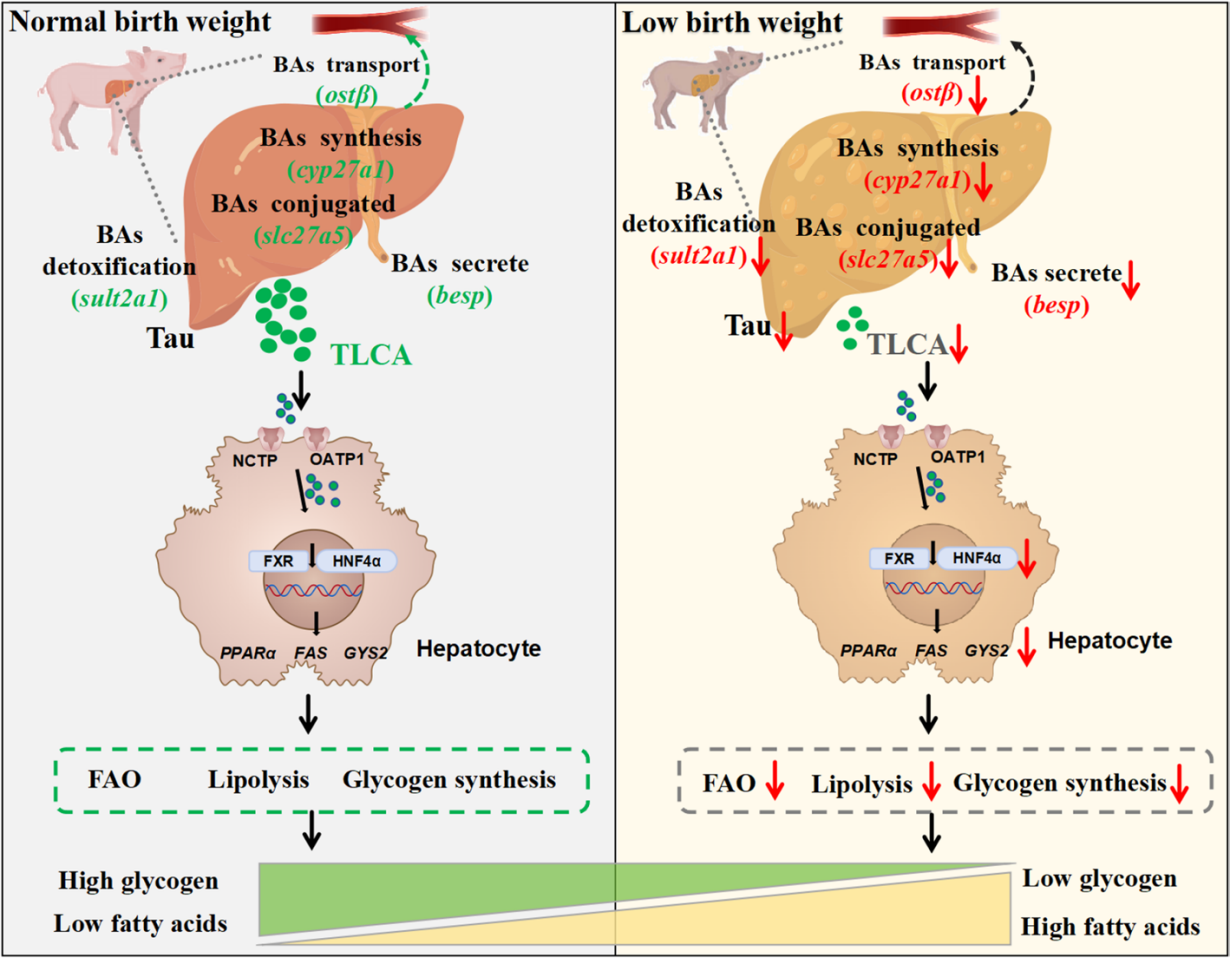
Mechanism of TLCA in alleviating hepatic glycolipid dysregulation in LBW piglets

In conclusion, our study found that the abnormal hepatic glucose and lipid metabolism in LBW fetal pigs and newborn piglets is associated with hepatic TLCA depletion and the downregulated FXR and HNF4α expression (Fig. 9). TLCA supplementation can activate FXR and HNF4α, thereby improving hepatic glucose and lipid metabolism as well as liver function in newborn LBW piglets.

## Supplementary Information

Additional file 1: Table S1. Ingredient and nutrient composition of experimental diets. Table S2. Primer sequences of target and reference genes in pig study. Table S3. Primer sequences of target and reference genes in Hepa 1-6 cell lines. Table S4. BA profiles of liver tissue. Fig. S1. LBW fetuses exhibit reduced hepatic glycogen stores and increased hepatic lipid accumulation. Fig. S2 and S3. Western blot images in this study.

## Data Availability

The datasets used and analysed during the current study are available from the corresponding author on reasonable request.

## Abbreviations

ALT: Alanine transaminase
AST: Aspartate transaminase
ATGL: Adipose triglyceride lipase
ACC: Acetyl-CoA carboxylase
BA: Bile acid
BAAT: Bile acid-CoA:amino acid N-acyltransferase
CA: Cholic acid
CDCA: Chenodeoxycholic acid
CPT2: Carnitine palmitoyltransferase 2
CYP7A1: Cytochrome p450 family 7 subfamily A member 1
DBIL: Direct bilirubin
FFAs: Free fatty acids
FXR: Farnesoid x receptor
FAS: Fatty acid synthase
Gly: Glycine
GYS2: Glycogen synthase 2
GCK: Glucokinase
G6PC: Glucose-6-phosphatase catalytic subunit 1
GLUT2: Glucose transporter 2
HSL: Hormone-sensitive lipase
HNF4α: Hepatocyte nuclear factor 4 alpha
HCA: Hyocholic acid
IL-6: Interleukin-6
IL-1β: Interleukin-1 beta
IUGR: Intrauterine growth restriction
LCA: Lithocholic acid
LBW: Low birth weight
NBW: Normal birth weight
PPARα: Peroxisome proliferator-activated receptor alpha
PPARγ: Peroxisome proliferator-activated receptor gamma
PYGL: Glycogen phosphorylase l
PEPCK2: Phosphoenolpyruvate carboxykinase 2
PC: Phosphatidyl choline
PFKL: Phosphofructokinase liver type gene
PKLR: Pyruvate kinase L/R
SLC27A5: Solute carrier family 27 member 5
SHP: Small heterodimer partner
SULT2A1: Sulfotransferase family 2A member 1
TBIL: Total bilirubin
Tau: Taurine
TNF-α: Tumor necrosis factor-α
TLCA: Taurolithocholic acid
