# Supplementary material for: Taurolithocholic acid alleviates hepatic glycolipid dysregulation in low-birth-weight piglets by activating HNF4α and FXR
Source: J Anim Sci Biotechnol. 2026 Aug 5;17:154. doi: 10.1186/s40104-026-01474-z (PMC13439904; doi:10.1186/s40104-026-01474-z)
Supplement: Supplementary file 1 [file 40104_2026_1474_MOESM1_ESM.docx]

**Table S1** Ingredient and nutrient composition of experimental diets (%, as-fed basis)

| **Items** | **Gestation** | **Lactation** |
| --- | --- | --- |
| Ingredients% |  |  |
| Corn | 57.79 | 51.17 |
| Wheat bran | 14.00 | 10.00 |
| Rice bran | 8.00 | 4.00 |
| Soybean meal | 11.00 | 15.45 |
| Soybean hull | 5.06 | - |
| Soybean oil | 0.55 | 2.53 |
| Soybean (heat-treated) | - | 8.00 |
| Fish meal Peru | - | 2.00 |
| Sucrose | - | 2.00 |
| Glucose | - | 2.00 |
| L-Lysine HCl（78.8%） | 0.16 | 0.13 |
| DL-Methionine (98%) | 0.04 | - |
| L-Threonine (98%) | 0.11 | 0.02 |
| L-Tryptophan (98%) | 0.03 | - |
| L-Valine (99%) | - | 0.08 |
| Dicalcium phosphate | 1.22 | 0.89 |
| Limestone | 1.12 | 0.89 |
| Sodium chloride | 0.40 | 0.40 |
| Premix^a^ | 0.52 | 0.44 |
| Total | 100 | 100 |
| Calculated analysis |  |  |
| DE, Mcal/kg | 12.77 | 14.24 |
| ME, Mcal/kg | 11.87 | 13.13 |
| Crude protein, % | 14.22 | 18.21 |
| Calcium, % | 0.70 | 0.66 |
| phosphorus, % | 0.81 | 0.71 |

^a^The premix (per kilogram of diet) provided the following: Gestation: Fe, 95mg; Cu, 46 mg; Zn, 100 mg; Mn, 54 mg and Se, 0.17 mg. vitamin A, 6,000 IU; vitamin E, 50 IU; vitamin D_3_, 1,200 IU; vitamin K_3_, 2.4 mg; vitamin B_1_, 1 mg; vitamin B_2_, 3.6 mg; vitamin B_6_, 1.8 mg; vitamin B_12_, 0.0125 mg; biotin, 0.24 mg; folic acid, 2 mg; niacin, 25 mg; pantothenic acid, 14 mg; preservative, 500 g; antioxidant, 200 g; mycotoxin adsorbent, 1,000 g; and choline chloride (60%), 1,300 g. Lactation: Fe, 100 mg; Cu, 25 mg; Zn, 125 mg; Mn, 35 mg; I, 0.2 mg; and Se, 0.3 mg. Vitamin A, 6,000 IU; vitamin A, 50 IU; vitamin D_3_, 1,200 IU; vitamin K_3_, 2.4 mg; vitamin B_1_, 1 mg; vitamin B_1_, 3.6 mg; vitamin B_6_, 1.8 mg; vitamin B_12_, 0.0125 mg; biotin, 0.24 mg; folic acid, 2 mg; niacin, 25 mg; pantothenic acid, 14 mg; preservative, 500 g; antioxidant, 200 g; mycotoxin adsorbent, 1,000 g; and choline chloride (60%), 1,000 g

**Table S2** Primer sequences of target and reference genes in pig study

| **Gene** | **Primer sequence (5'→3')** | |
| --- | --- | --- |
|  | **Forward** | **Reverse** |
| *BAAT* | TCCCCATTGAAAAGGCCCAG | GTTCTGTGGCTTGTTGTGCG |
| *FGF19* | TGAGTACCGTGGCGATCAAG | GCGGATCTCCTCCTCGAAAG |
| *BESP* | CGCAGCGTGAAGAAATGTGG | AAAACCGAAACAGTTGAAAGAGGC |
| *CYP8B1* | GGAGCGACTTGGCTTTCTCT | GCAGGCGGAGGAGTTATTCA |
| *HNF4α* | GCTTCTTTCGGAGGAGTGTG | TTGACCTGCGAGTGCTGAT |
| *FXR* | TATGAACTCAGGCGAATGCCTGCT | ATCCAGATGCTCTGTCTCCGCAAA |
| *CYP7A1* | GAAAGAGAGACCACATCTCGG | GAATGGTGTTGGCTTGCGAT |
| *CYP27A1* | ACTGAAGACCGCGATGAAAC | CAAAGGCGAATCAGGAAGGG |
| *CYP4A21* | TTTTCCCGCTTGAGGAGTGC | ACTCGGTCTGTGTGTTGATGGA |
| *MRP2* | TGCAAGTACGGACCAGTGTC | AACGGTGTACTGCTTCCTGG |
| *MRP3* | TGGACAAAGGGACAATAGCTGAGT | TGGCCATCCCGTAGAAGATG |
| *OSTβ* | GAAATCCAAAGACGCTGCCA | CCCTTAGGATGGTCAGGTTGT |
| *OATP1* | TGACACTGGATCGGTGAACA | AGGAATGGCAGTGAGCACAT |
| *SULT2A1* | CCATGCGAGACAAGGAGAAC | CATGACCTGGAAGGAGCTGT |
| *SLC27A5* | CATTCAGTCCGCCTGGCTAT | AAGCCGACATTGCCTTCTGT |
| *GYS2* | AATTCTGTGGGAAGCCCAGC | GCAGGGATTCAGAGCTGGTA |
| *GCK* | CCGACTTCCTGGACAAGCAT | ATCGTGGCCACAGTGTCATT |
| *PFKL* | ACTCCCTTCGACCGGAACTA | TGCTCAAAGTCGGTGTCCTA |
| *PKLR* | CCCACTGAAGTCACCGCTAT | GAGGAAGCCACGGAGTTTT |
| *PYGL* | ATCGGGGAGGACTACGTGAA | CTCGGAGGAAGACATCGTCG |
| *G6PC* | TGAACGTCTGTCTGTCACGA | ATACTTCTTGAGGCTGGCGT |
| *PC* | GGACTTCACTGCCACCTTTG | GCTCCACCTCAAACTCCTCT |
| *GLUT2* | GGTTCATGGTGGCCGAGTT | ATTGCGGGTCCAGTTGC |
| *HSL* | AGTGCCTATGCTGGCGGGGA | CCCAGGCGGAGGTCTCGGAA |
| *ATGL* | GGCGGAACGGCCTCCTGAAC | TTGGCTCCGGCCCTCTCCTC |
| *PPARγ* | AAGACGGGGTCCTCATCTCC | CGCCAGGTCGCTGTCATCT |
| *ACC* | AGCAAGGTCGAGACCGAAAG | TAAGACCACCGGCGGATAGA |
| *FAS* | CTACGAGGCCATTGTGGACG | AGCCTATCATGCTGTAGCCC |
| *SREBP-1c* | AAGCGGACGGCTCACAATG | GCAAGACGGCGGATTTATTCA |
| *CPT1α* | GATTATCCACCAGCCAGACCC | CCCCACAATAATGTACGACACC |
| *HMGCS2* | GGACCAAACAGACCTGGAGA | ATGGTCTCAGTGCCCACTTC |
| *PPARα* | GAGTTCGCCAAGTCCATCC | CCGTCCTTGTTCATCACAGAG |
| *ACTB* | CACCTTCTACAACGAGCTGC | TCATCTTCTCACGGTTGGCT |

**Table S3** Primer sequences of target and reference genes in Hepa 1-6 cell lines

| **Gene** | **Primer sequence (5'→3')** | |
| --- | --- | --- |
|  | **forward** | **reverse** |
| *HSL* | TGGTGACACTCGCAGAAGAC | GATGGCAGGTGTGAACTGGA |
| *GLUT2* | TGAGTTCCTTCCAGTTCGGC | ATTCCTGAGAACTGCTGGGC |
| *GYS2* | TCCTCAGTACCACCTTCC | AGCCTCCTCTTCCTCATC |
| *ACTB* | GGAGATTACTGCCCTGGCTCCTA | GACTCATCGTACTCCTGCTTGCTG |
| *SHP* | CGATCCTCTTCAACCCAGATG | AGGGCTCCAAGACTTCACACA |
| *CYP7A1* | AGCAACTAAACAACCTGCCAGT | ACTAGTCCGGATATTCAAGGATGCA |
| *FXR* | CGTGACTTGCGNCAAGTGACC | CCANGACATCAGCATCTCAGC |
| *HNF4α* | GGGCTGGCATGAAGAAGGAAGC | CCATGCCGAAGAGCTTGATGAAC |
| *PPARα* | GATGCACTGCTATGAGCACTT | AGAGGTCCACAGAGCTGATCC |
| *ACC* | GGGAATACCTGTGGGAGTAGT | GCTGGATTATCTTGGCTTCA |
| *FAS* | CATCCGGTCAGAGAGTACTTTT | TAGGGTTATGATGCTCTTCACC |
| *CPT1* | TATGTGAGGATGCTGCTTCC | CTCGGAGAGCTAAGCTTGTC |
| *PEPCK* | TGCGGATCATGACTCGGATG | AGGCCCAGTTGTTGACCAAA |

**Table S4** Bile acid profile of liver tissue, nmol/g

|  | **Group** | | ***P*-value** |
| --- | --- | --- | --- |
|  | **NBW** | **LBW** |  |
| C4 | 0.36 ± 0.03 | 0.42 ± 0.06 | 0.084 |
| TwMCA | 0.43 ± 0.10 | 0.58 ± 0.34 | 0.377 |
| TaMCA | 0.17 ± 0.05 | 0.21 ± 0.14 | 0.515 |
| TbMCA | 0.38 ± 0.10 | 0.57 ± 0.46 | 0.377 |
| THCA | 3.96 ± 1.80 | 4.01 ± 2.50 | 0.969 |
| TCA | 3.11 ± 1.65 | 2.49 ± 0.70 | 0.460 |
| THDCA | 23.12 ± 16.54 | 9.83 ± 7.09 | 0.130 |
| TCDCA | 22.28 ± 14.10 | 10.37 ± 5.53 | 0.109 |
| TDCA | 0.10 ± 0.06 | 0.07 ± 0.03 | 0.289 |
| TLCA | 0.27 ± 0.13 | 0.06 ± 0.03 | 0.005 |
| wMCA | 0.31 ± 0.12 | 0.32 ± 0.08 | 0.967 |
| aMCA | 0.41 ± 0.13 | 0.42 ± 0.06 | 0.904 |
| bMCA | 0.86 ± 0.50 | 0.85 ± 0.42 | 0.972 |
| HCA | 0.11 ± 0.06 | 0.23 ± 0.16 | 0.150 |
| AlloCA | 0.31 ± 0.14 | 0.32 ± 0.06 | 0.876 |
| CA | 0.80 ± 0.42 | 1.07 ± 0.61 | 0.434 |
| NorCA | 0.03 ± 0.00 | 0.03 ± 0.00 | 0.740 |
| GHCA | 25.16 ± 10.38 | 35.71 ± 22.06 | 0.356 |
| GCA | 5.14 ± 2.31 | 7.73 ± 2.19 | 0.099 |
| GHDCA | 37.85 ± 22.52 | 27.49 ± 17.64 | 0.437 |
| GCDCA | 24.66 ± 12.96 | 18.55 ± 8.53 | 0.399 |
| GDCA | 0.27 ± 0.08 | 0.24 ± 0.06 | 0.545 |
| GLCA | 0.28 ± 0.13 | 0.08 ± 0.03 | 0.010 |
| muroCA | 0.74 ± 0.27 | 1.30 ± 0.57 | 0.079 |
| bHDCA | 0.08 ± 0.03 | 0.10 ± 0.03 | 0.216 |
| UDCA | 0.04 ± 0.02 | 0.04 ± 0.01 | 0.787 |
| HDCA | 0.63 ± 0.29 | 1.15 ± 0.40 | 0.041 |
| bDCA | 0.03 ± 0.02 | 0.03 ± 0.02 | 0.508 |
| CDCA | 0.23 ± 0.10 | 0.37 ± 0.14 | 0.111 |
| DCA | 1.78 ± 1.41 | 1.48 ± 0.77 | 0.684 |
| NorDCA | 0.39 ± 0.05 | 0.40 ± 0.06 | 0.800 |
| isoalloLCA | 0.03 ± 0.02 | 0.02 ± 0.01 | 0.676 |
| isoLCA | 0.01 ± 0.01 | 0.01 ± 0.00 | 0.780 |
| LCA | 0.11 ± 0.06 | 0.10 ± 0.03 | 0.687 |
| LCA-3S | 0.45 ± 0.06 | 0.46 ± 0.06 | 0.765 |
| dehydroLCA | 0.01 ± 0.00 | 0.01 ± 0.00 | 0.736 |
| 6-KetoLCA | 0.09 ± 0.03 | 0.12 ± 0.06 | 0.346 |
| 7-KetoLCA | 0.32 ± 0.04 | 0.33 ± 0.04 | 0.736 |
| 12-KetoLCA | 0.41 ± 0.11 | 0.39 ± 0.05 | 0.847 |
| apoCA | 0.06 ± 0.01 | 0.06 ± 0.03 | 0.935 |
| 7-DHCA | 0.20 ± 0.16 | 0.19 ± 0.09 | 0.953 |
| 12-DHCA | 0.03 ± 0.01 | 0.03 ± 0.02 | 0.770 |
| 3-DHCA | 0.08 ± 0.01 | 0.08 ± 0.01 | 0.273 |
| CA-3-S | 0.47 ± 0.20 | 0.43 ± 0.11 | 0.724 |
| Sulfate-GCA | 0.14 ± 0.01 | 0.19 ± 0.02 | 0.001 |
| Conjugated BAs | 147.32 ± 79.90 | 118.18 ± 63.20 | 0.537 |
| Unconjugated BAs | 9.37 ± 3.77 | 10.74 ± 1.58 | 0.469 |
| TBAs | 156.61±78.80 | 128.92±62.87 | 0.552 |

**Fig. S1** LBW fetuses exhibit reduced hepatic glycogen stores and increased hepatic lipid accumulation. **A** Concentrations of primary BAs, secondary BAs, and TBAs in liver tissue. **B** Concentrations of primary BAs, secondary BAs, and TBAs in liver tissue. **C** Serum and liver sult2a1 Concentrations

**Fig. S2** Blots image in animal study. **A** Relative expression levels of FXR and HNF4α proteins normalized to β-actin in the liver tissue. **B** Protein expression levels of SLC27A5 and CYP27A1 normalized to β-actin in the liver. **C** Relative protein expression levels of FXR and HNF4α normalized to β-actin in the liver

**Fig. S3** Blots image in Hepa1-6 cells. Relative protein expression levels of CYP7A1, FXR, HNF4α, and SHP normalized to β-actin
